# Correlation between the prevalence of herniation pits and the alpha angle of the hip: computed tomography evaluation in healthy Chinese adults

**DOI:** 10.1186/1471-2474-14-288

**Published:** 2013-10-09

**Authors:** Zhe Guo, Li Xu, Yong-bin Su, Xiao-guang Cheng

**Affiliations:** 1 Department of Radiology, Beijing Jishuitan Hospital, 31 Xinjiekou East Street, Xicheng District, Beijing 100035, China

**Keywords:** Herniation pits, Prevalence, Femoral neck, Alpha angle, Computed tomography

## Abstract

**Background:**

Herniation pits (HPs) commonly develop over time at the femoral head–neck junction in adults, but their cause is still under debate. The purpose of study reported here was to investigate the correlation between the prevalence of HPs of the femoral neck and the alpha angle of the hips of healthy Chinese adults, by using computed tomography (CT).

**Methods:**

Six hundred and seventy Chinese adults (representing 1145 hips) who had no known diseases affecting the proximal femur and had no symptoms of femoroacetabular impingement underwent a 64-slice CT scan for medical purposes that included the hip in the scan range. Their CT data were analyzed for the prevalence of HPs in the femoral necks and for hip alpha angles.

**Results:**

The overall prevalence of femoral-neck HPs was 12.5% (143 of 1145 hips). The prevalence in the left versus right femoral necks was 12.1% (69 of 569 hips) versus 12.8% (74 of 576 hips). There was no statistically significant difference between the two sides (χ^2^ = 0.136; p = 0.712). The prevalence of HPs was greater in men than in women (15.9% vs 7.7%; p < 0.01) and greater in adults older than 30 years than in adults younger than 30 years (χ^2^= 14.547; p < 0.01). The alpha angles were greater in the 143 proximal femora with HPs than in the 1002 without pits (39.95° ± 6.01° vs 37.97° ± 5.14°; p < 0.01).

**Conclusions:**

The prevalence of HPs of the femoral neck in healthy adults was 12.5%, and the prevalence was greater in men than in women. There is a correlation between the prevalence of HPs and the contour of the femoral head–neck junction. The formation of pits may be attributed to the combination of degeneration and morphologic variances in the femoral head–neck junction.

## Background

A common change in adults over time is the development of herniation pits (HPs) of the femoral neck, below the cortex of the femoral head–neck junction. This phenomenon was first reported in 1982 by Pitt *et al.*[1]. Radiographic imaging can detect relatively larger HPs, and multidetector computed tomography (CT) can detect small HPs [2-4]. The typical manifestation of HPs is in the form of a round, hypodense region (with a diameter of <10 mm, a sharp border, and peripheral sclerosis) located below the anterior cortex of the femoral neck. Some HPs have a linear fissure that is in contact with the joint cavity. The cause of HPs is unclear [3,5]. In the 20th century, HPs were described as fibrocystic lesions consisting of synovial tissue [1]. However, since the beginning of the 21st century, as the concept of femoroacetabular impingement (FAI) has become popular, HPs have been considered to be an indicator of FAI [6-9]. Leunig *et al.* reported a high prevalence of HPs in patients with FAI (39 of 117 hips) [6]. The alpha angle is an important index for evaluating the contour of the femoral head–neck junction [10-12]. An alpha angle >50° may indicate a cam type of FAI [10]. Panzer *et al.* reported that the alpha angle is 10% larger in patients with FAI and HPs, and this difference was significant [13]. We investigated the correlation between the prevalence of HPs of the femoral neck and the alpha angle of the hip in healthy Chinese adults.

## Methods

### Study participants

Between September 2009 and March 2010, 670 Chinese adults who had no known diseases affecting the proximal femur and no symptoms of FAI underwent a 64-slice CT scan for medical purposes (e.g., pelvic diseases, trauma); the hip was included in the scan range. The exclusion criteria included fracture, malformation, and tumor in the proximal femur; a bony bump at the femoral head–neck junction; and overcoverage of the acetabulum. There were 393 male and 277 female study participants. Their ages ranged from 16 to 92 years; the average age was 46 years (Table 1). The epiphyseal lines of all proximal femora had closed. Approval for our study was obtained from the Research Ethics Committee, Beijing Jishuitan Hospital, China, and the study protocol was in compliance with the Helsinki Declaration.

**Table 1:** Distribution of gender and age of study participants

**Gender**	**Age group (years)**							**Total**
	**16–20**	**21–30**	**31–40**	**41–50**	**51–60**	**61–70**	**>71**	
M	25	75	72	82	65	33	41	393
F	20	32	48	55	55	21	46	277
Total	45	107	120	137	120	54	87	670

### Examination technique

**Figure 1:**
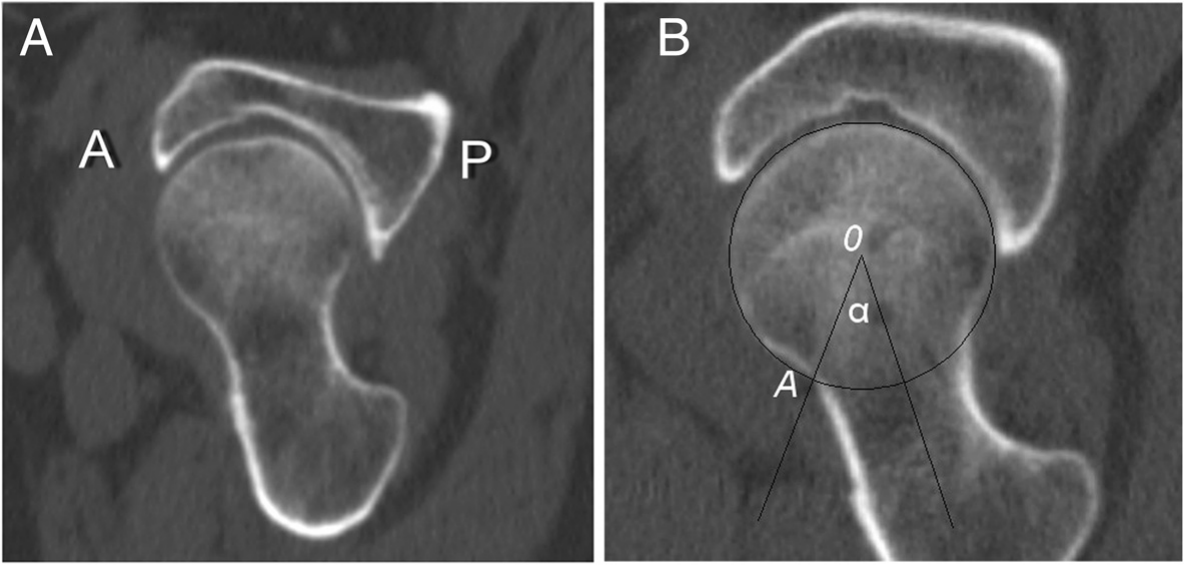
**Reconstructed CT image of proximal femora and measurement of the alpha angle. (A)** View of the oblique axial plane parallel to the axis of the femoral neck. **(B)** Measurement of the alpha angle; see the text for definitions of the various points.

All CT scans were obtained using a 64-slice CT scanner (Aquilion 64, Toshiba, Beijing, China) with the following parameters: tube voltage, 120 kV; tube current, 200 to 350 mA; pitch, 1.0 mm; slice thickness, 0.5 mm; matrix, 521 × 512 pixels. All images were reconstructed using standard algorithms. All volume data were then processed on the workstation connected to the CT scanner. Transverse plane images and oblique axial plane images parallel to the axis of the femoral neck were reconstructed by a radiologist. The slice thickness and slice interval were both 1 mm. For all images, the bone window width was 1500 Hounsfield units (HU) and the bone window level was 350 HU (Figure 1A). Two musculoskeletal radiologists (one with 7 years’ experience and the other with 5 years’ experience) assessed whether HPs existed. When their individual results were not consistent, they reached an agreement through discussion. Images of the oblique axial plane through the axis of the femoral neck were chosen to measure the alpha angle, using AutoCAD 2006 software (Autodesk, San Rafael, CA, USA).

### Image analysis

HPs were defined as cystic lesions located below the anterior cortex of the femoral neck and having a diameter of ≥2 mm [1,13]. The alpha angles of the hips were measured according to the method developed by Notzli *et al.*[10], in which the anterior extent of the concavity of the femoral neck is defined as the point (point *A* in Figure 1B) at which the distance from the cortex to the center of the femoral head (point *0* in Figure 1B) first exceeds the radius of the femoral head. The angle formed between the axis of the femoral neck and the line connecting the center of the femoral head to point A is the alpha angle (α in Figure 1B).

### Statistical analysis

Statistical analysis was performed by using SPSS software (version 15.0; IBM, Armonk, NY, USA). The alpha angles of the femora of study participants were nearly normally distributed. The differences in prevalence of HPs between the two sides, between men and women, and among different age groups were analyzed using the chi-square test, and the difference between the alpha angles of groups with and without HPs was analyzed using the *t*-test.

## Results

**Figure 2:**
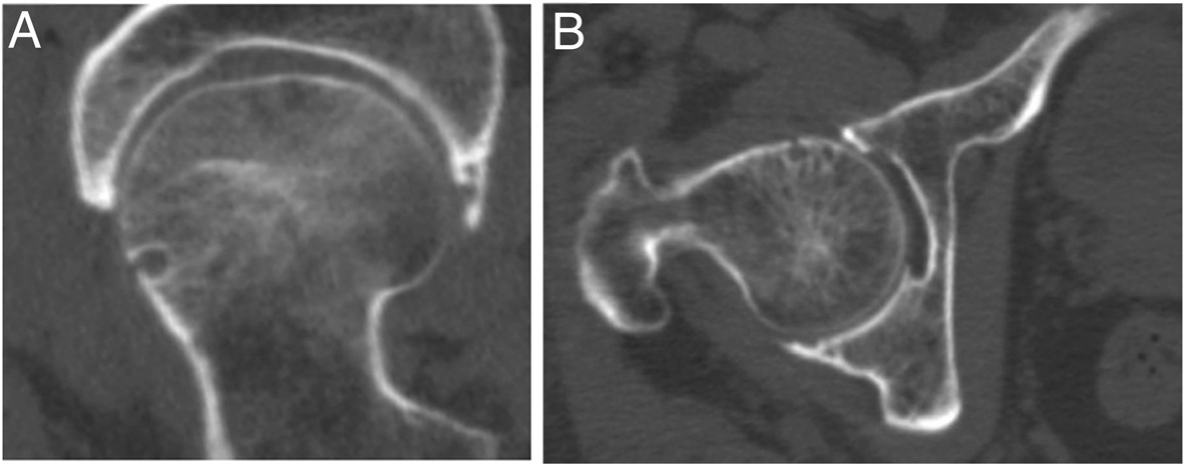
**Computed tomography images of herniation pits. (A)** Oblique axial and **(B)** transverse computed tomography images of the right hip of a 85-year-old man, showing herniation pits within the proximal femora.

All data for the 670 study participants were analyzed and 195 hips were excluded because of fracture, malformation, and tumor in the proximal femur. HPs were present in 143 of the 1145 proximal femora (Figure 2), a prevalence of 12.5%. The prevalence of HPs in the left and right femoral neck was 12.1% (69 of 569 hips) and 12.8% (74 of 576 hips), respectively. There was no statistically significant difference between the two sides (χ^2^ = 0.136; p = 0.712).

The prevalence of HPs was greater in men than in women (χ^2^ = 17.014; p < 0.01), being 15.9% (107 of 675 hips) in men and 7.7% (36 of 470 hips) in women. The participants were also divided into 7 groups by age so that the prevalence of HPs for the different age groups could be compared. We found statistically significant differences in prevalence among the different age groups (χ^2^ = 18.012; p < 0.01) (Table 2). There were no statistically significant differences in the prevalence of HPs among the five groups of participants older than 30 years (χ^2^ = 2.399; p = 0.121), but those groups had a much greater prevalence of HPs than did the groups of participants younger than 30 years (χ^2^ = 14.547; p < 0.01).

**Table 2:** Comparison of the prevalence of herniation pits in proximal femora among age groups

**Parameter**	**Age group (years)**							**Total**
	**16–20**	**21–30**	**31–40**	**41–50**	**51–60**	**61–70**	**>71**	
Femurs	77	187	211	229	200	92	149	1145
Herniation pits	3	12	35	32	30	15	16	143
Prevalence (%)	3.9	6.4	16.6	14.0	15.0	16.3	10.7	12.5

The mean value of the alpha angle for all proximal femora was 38.20° ± 5.33°. In comparison, the mean value of the alpha angle of the 143 femora with HPs was 39.95° ± 6.01°. The mean value of the alpha angle for the 1002 femora without HPs was 37.97° ± 5.14°. The difference between the alpha angles for both groups reached statistical significance (*t* = 3.720; p < 0.01).

## Discussion

The cause of HPs is unclear. Most authors believe that HP formation is correlated with high pressure in the joint capsule and the friction between the joint capsule and the anterior cortex of the femoral neck [3,5,14,15]. It has been recently reported that FAI may be a cause of HPs [6-9,13,16]. In a prior study using radiographic imaging, the prevalence of HPs in healthy adults was found to be approximately 5% [1]. With the widespread use of CT, however, a greater number of HPs are now being detected. However, the results of different studies vary. To our knowledge, our study has the largest sample of HPs to date. However, the prevalence of HPs in our study is significantly lower than the prevalence reported in another study that involved 400 hips and used a similar methodology (12.5% vs 26.7%) [13]. The difference in prevalence may be a result of the differences in the selection criteria for participants for each study. In addition, it has been reported that the incidence of primary hip osteoarthritis is significantly lower in Asians than in whites [17], a finding that is similar to the distribution pattern for the prevalence of HPs. Thus,whether differences in ethnic groups and lifestyles in different regions affect the prevalence of HPs should be studied further.

The prevalence of HPs is significantly greater in men than in women. This may be because in general, men take part in more manual labor and physical exercise than women do [18,19]. Therefore, more friction occurring between the joint capsule and the anterior cortex may contribute to the formation of HPs. It is also possible that the higher prevalence of HPs in women is related to postmenopausal osteoporosis or other bone changes, and this possibility should be further investigated.

We found statistically significant differences in the prevalence of HPs among age groups. There was a much greater prevalence of HPs in participants older than 30 years than in those younger than 30 years (14.5% vs 5.7%), indicating that the turning point for increased prevalence is sometime after the age of 30 years. Although there is no research supporting any theory for the distribution of HPs in different age groups, we believe that the prevalence of herniation increases with age because participation in manual labor and physical exercise peaks in the thirties, then plateaus after age 40 years because of lifestyle changes, including decreased participation in these activities [19].

The alpha angle is an important index of the morphology of the femoral head–neck junction. In patients with cam-type FAI, the normal offset of the femoral head–neck junction disappears and the alpha angle obviously increases [20]. Some reports have indicated a correlation between HPs and FAI [6-9,13,16]. According to our study, the difference between both groups with or without HPs in the alpha angles reached statistical significance, which may illustrate the correlation between HPs and the morphology of femoral head–neck junction. Our results are in accord with the study of Panzer *et al.* in this respect [13]. A larger alpha angle may lead to less space and more impingement between the anterior cortex of the femoral neck and the acetabular rim or joint capsule, and accordingly induce the formation of HPs.

Our study had some limitations. First, it exclusively addressed the relationship between the alpha angle and the prevalence of HPs and did not take acetabular morphology into consideration. Second, it did not specify the kinds of manual labor and physical exercise that would put study participants at risk for the development of HPs.

## Conclusions

The prevalence of HPs in healthy Chinese adults is 12.5%. Men have a greater prevalence of HPs than women do, and people older than 30 years have a greater prevalence of HPs than do people younger than 30 years. There is a correlation between the development of HPs and the morphology of the femoral head–neck junction. The formation of HPs may be attributed to the combination of degeneration and morphologic variances in the femoral head–neck junction.

## Abbreviations

CT: Computed tomography; FAI: Femoroacetabular impingement; HP: Herniation pit.

## Competing interests

The authors declare that they have no competing interests.

## Authors’ contributions

ZG initiated the study, reviewed the data, reconstructed and analyzed the images, and drafted and revised the manuscript. XGC reviewed the data, discussed the study, and revised the manuscript. LX and YBS reviewed the data, discussed the study, analyzed the images, and provided valuable comments on the manuscript. All authors reviewed the manuscript and approved the final version.

## Pre-publication history

The pre-publication history for this paper can be accessed here:

http://www.biomedcentral.com/1471-2474/14/288/prepub
